# A qualitative evaluation of a STEAM (Science, technology, engineering, arts, and mathematics) peer education intervention on teenagers’ knowledge, confidence, and advocacy for HPV vaccine communication

**DOI:** 10.1080/21645515.2026.2745405

**Published:** 2026-10-28

**Authors:** Carol Gray Brunton, Iain Macdonald, Céline Healy, Richard Firth, Alexandra McDermott, Eva Malone

**Affiliations:** a Previous School of Health & Social Care, Edinburgh Napier University, Edinburgh, UK; b Department of Design Innovation, Maynooth University, Co. Kildare, Ireland; c Department of Education, Maynooth University, Co. Kildare, Ireland; d School of Arts and Creative Industries, Edinburgh Napier University, Edinburgh, UK; e Glanbia Business Services, Dublin, Ireland; f School of Applied Sciences, Edinburgh Napier University, Edinburgh, UK

**Keywords:** HPV vaccine, immunology, dramatic inquiry pedagogy, Art and Design, adolescents, peer education, co-design, vaccine hesitancy, psychology

## Abstract

Vaccine hesitancy is a rising global phenomenon. Ireland experienced a crisis in confidence and vaccine decline for the Human Papillomavirus (HPV) vaccine in 2015, with rapid national efforts to address concerns and improve uptake rates. We developed a unique STEAM (Science, Technology, Engineering, Arts and Mathematics) peer education intervention through an interdisciplinary team (immunologist, educationalist, designer, filmmaker, and psychologist) to address vaccine hesitancy and utilize peer education, which is promising but underused in the literature. We aimed to evaluate the effect of our STEAM intervention on Irish teenagers’ knowledge and confidence in advocacy for the HPV vaccine to their younger peers facing their HPV vaccine offer. We used qualitative methods consisting of 12 focus group discussions with teens (n = 53), one qualitative in-class evaluation among younger peers (n = 35), and knowledge exit slips (n = 300) to evaluate teenagers’ knowledge, confidence, advocacy, and younger peers’ knowledge. Data were analyzed inductively using reflexive thematic analysis. Our overall egalitarian theme indicated democratic participation by teenagers with three related themes that were compatible with behavior as a vaccine advocate. Teenagers reported positive changes overall in HPV knowledge, confidence, and advocacy beliefs following the intervention. Younger peers could recall HPV facts and knowledge during in-class evaluations and exit slips. We conclude that scaffolding through our STEAM intervention enabled Irish teenagers to feel empowered to act as vaccine advocates. Peer education within our intervention shows promise in raising HPV awareness and knowledge and could be explored in wider contexts.

## Introduction

Vaccine hesitancy, delays, or refusal to be vaccinated is a global phenomenon that depends on local context.^1^ European contexts are particularly susceptible to vaccine hesitancy,^2^ which has increased in many countries since the COVID 19 pandemic.^3^ The Human Papillomavirus (HPV) vaccine, introduced to protect against common oncogenic strains of the sexually transmitted HPV virus implicated in cervical, anogenital, and oropharyngeal cancers that can affect women and men, is commonly delivered to young people before sexual debut.^4^ Cancer prevention through the HPV vaccine has been increasingly demonstrated. For example, in Scotland, no new cases of invasive cervical cancer have been identified in a cohort of young women vaccinated in a national program.^5^

Like many nations, Ireland offers the HPV vaccine in a national school-based program in the first year of secondary school (aged 12–13 years), available for girls since 2010 and for boys since 2021. Initially, HPV vaccine uptake rates were over 80%; however, in 2015, there was a crisis of confidence spread through social media. Ireland was not the only country affected, and similar concerns were observed in Denmark and Japan. Ireland responded rapidly to address such concerns,^6^ and HPV vaccine uptake rates have recovered to 74% by 2023/2024.^7^ Lower HPV vaccine uptake rates have challenged the European Cancer Organization (ECO), and the World Health Organization (WHO) calls for a 90% HPV vaccine uptake rate among girls to eliminate cervical cancer and to reach boys.^8,9^ Face-to-face educational interventions have been identified as effective in increasing vaccination intentions among children, adolescents, parents, and young adults.^10^ Multicomponent interventions, including a health provider strategy, appear to increase HPV vaccination uptake when tailored to local contexts.^10^ Many interventions for improving HPV vaccination target parents and healthcare providers. It is also important to include adolescents and young people, the vaccine recipients, in HPV vaccine interventions.^11^ Boys/young men should also be included in HPV vaccine interventions, given recommendations for gender-neutral vaccine campaigns using tailored approaches that focus on wider cancers.^11–14^

Previous studies have indicated low HPV knowledge and awareness among European adolescents,^15^ including parents of boys.^16^ Peer education may be a promising intervention for addressing vaccine hesitancy. This refers to peers, adolescents, or young people educating their peers of similar ages about vaccines building rapport with the audience, increasing knowledge, and fostering positive attitudes toward potential behavior change, although support, planning, and resourcing are careful considerations.^11,17^ Vaccine acceptance through peer education was more effective when peer educators and health experts worked together, particularly if it was more than a one-off intervention.^17^ While promising, further research is needed to identify key components for successful peer education implementation for different peer groups, contexts, and vaccine-hesitant groups.^17^

We developed a novel Science, Technology, Engineering, Arts and Mathematics (STEAM) Peer Education intervention that is linked to the Irish secondary school curriculum. The multidisciplinary team comprised an educationist skilled in language and drama, an immunologist, a filmmaker, a designer, and a psychologist. The intervention aimed to address gender-neutral vaccine hesitancy, use storytelling, emphasize the normal functioning of the immune system, co-create elements with participants, and use peer education. The initial approach for this intervention was refined from a previous iteration.^18^

The rationale for the multidisciplinary approach was to facilitate learning and support HPV peer educators informed by design thinking,^19^ dramatic inquiry pedagogy,^20^ science, and behavioral evidence. Design thinking approaches utilize a range of creative media, including drama, storytelling, posters, cartoons, video, and filmmaking, and can facilitate access to complex scientific topics,^21,22^ such as comic book creation,^23^ storytelling^24,25^ and videography.^26,27^ In HPV communication, creative approaches have been used effectively to reach underserved communities, including young men with an intellectual disability, through activity-based approaches^28^ and filmmaking with ethnic minority parents and young people.^29,30^

Dramatic inquiry pedagogy activities ensure that learning is social, with modeling opportunities integrated to enable new learning for peers in small groups using movement-based activities.^20^ Immunology expertise provides an essential understanding of how the immune system functions and how HPV vaccines can be used by the immune system to protect against HPV. This was supported by Irish Cancer Society (ICS) researchers in accessible and interactive learning slots/videos. An evaluation of the intervention was conducted by the psychologist and team. This STEAM peer education intervention utilized creative outputs co-designed with teenagers and experts to advocate and communicate the HPV vaccine to younger peers facing their HPV vaccine offer. This study aimed to explore teenagers’ knowledge, confidence, and advocacy for communication about the HPV vaccine to their younger peers’ and younger peers’ knowledge recall.

## Materials and methods

### Study setting and participants

Five secondary schools from two counties in Ireland were purposively selected, including two schools with rural and diverse socioeconomic backgrounds and existing working relationships with the University’s Department of Education (Maynooth University). In Ireland, the national school-based HPV vaccine program was extended to boys in 2021, and participants included both girls and boys. This was the first cohort of boys to be offered the HPV vaccine. Four were mixed co-ed schools, and one was an all-boys school.^1^ Participants opted into the intervention at age 15–16, in their transition year (TY) at one of the five secondary schools (n = 44).

### STEAM intervention

The STEAM intervention consisted of three phases from March 2023 to June 2024. Table 1 summarizes these phases.

**Table 1.** The STEAM peer education intervention.

Phase	STEAM Intervention description
1 STEAM Workshops (March 2023)	University campus STEAM Workshops with co-design of creative materials over 3 days with teens (aged 15–16) (n = 44) from five schools
2 Creative outputs for Peer Education (May–June 2023 and March 2024)	Development and co-design of creative outputs with teens (aged 15–16 year) for younger peers and subsequent peer education in primary schools and secondary schools - with 4 teens for younger peers (aged 11–12 year) at two primary schools - and with 17 teens for younger peers (aged 12–13 year) at two secondary schools - knowledge recall among younger peers (11–13 year) (n = 275) pre-vaccine offer
3 Knowledge recall (June 2024)	School visits by university academics to assess knowledge recall of STEAM project by the younger peer (aged 12–13 year), following their HPV vaccination offer in secondary schools (n = 25)

### Phase 1: STEAM workshops

STEAM workshops (n = 2) were held on a university campus with teenagers from secondary schools, with a multidisciplinary team of university academics (design, education, immunology, and psychology), and researchers from the Irish Cancer Society. Teenagers worked collaboratively in small groups from different schools for over 3 days (12 hours) through activities supporting the co-design of creative outputs on HPV vaccination. Dramatic inquiry pedagogy^20^ fostered a collaborative sense of community and facilitated reflections to visually represent their current levels of knowledge and understanding of the HPV vaccine and immunology. Self-and-group reflective activities developed and shared ideas about HPV and the vaccine, including the emotional responses to vaccination. These activities were supplemented by expert information on immunology, HPV, HPV infection, and associated cancers. Multimedia approaches were encouraged so that teenagers could consolidate, develop, and present their knowledge throughout the workshops. These included storyboarding, posters, improvisation, role-play, pictures, diagrams, movement, and sound, which facilitated communication between teens who were not familiar with each other prior to the workshops. The creative activities allowed the development of ideas, and teenagers explored what information was important for sharing with their younger peers in discussions with experts. The end-workshop output was student-made films with persuasive content about HPV vaccine promotion.

### Phase 2: creative outputs for peer education

Phase 2 took place at 2 time points, in June 2023 and March 2024: teenagers used their co-designed creative outputs (films and posters) to deliver peer education to younger peers. This peer education was co-delivered with experts to two primary schools and two secondary schools prior to their HPV vaccine offer, using their outputs, role-play, and an immunology presentation to support HPV communication.

### Phase 3: knowledge recall post-vaccination offer

Phase 3 took place in June 2024 as school visits by university experts post-vaccination offers to identify knowledge recall by younger peers (aged 12–13 years) from Peer Education (Phase 2) after a longer period. Maintaining contact with schools and finding time in their timetables to undertake this phase was challenging because of the delay in the National Immunization Office’s vaccine roll-out in schools, which delayed and limited this phase.

### Qualitative evaluation data collection

Qualitative evaluation was appropriate for this exploratory intervention. The overall aim was to explore teenagers’ knowledge, confidence, and advocacy during the STEAM intervention and their younger peers’ knowledge recall. The data were collected iteratively during each phase. Table 2 illustrates the main qualitative data collection methods used for each phase, comprising focus group discussions, in-class evaluation, and open-exit slips which were open surveys for younger peers’ responses to a single question (*If you were to tell a friend who was absent today about HPV and the vaccine, what would be the main points that you’d highlight for them? Think about it for a moment then write the keywords that you would include with your summary for them and if you want, draw a picture to accompany them*).

**Table 2.** Qualitative evaluation data collection for the STEAM peer education intervention.

STEAM Phase	Data Collection	Participants
Phase 1	Qualitative focus group discussions were used to explore teens’ STEAM experiences, HPV knowledge, confidence and advocacy following the STEAM workshops from a subset of teens in their working groups	Teens (n = 32) from 7 focus groups
Phase 2	Qualitative focus groups were used to explore teen’s HPV knowledge, confidence and advocacy following presentation of co-designed workshop to peers in their working groups A Qualitative in-class evaluation was used to explore HPV knowledge recall from younger peers after the STEAM Peer education at primary school before their HPV vaccine offer Brief open exit slips were used to explore HPV knowledge recall from younger peers after the STEAM Peer Education before their vaccine offer	Teens (n = 21) from 5 focus groups Younger peers (n = 35) from 1 in-class evaluation Younger peers (n = 275 open response slips)
Phase 3	Brief open exit slips were used to explore HPV knowledge recall from younger peers after the STEAM Peer Education from secondary school after their HPV vaccine offer	Younger peers (n = 25 open response slips)
Total		12 Focus groups with teens (n = 53) 1 Qualitative in-class evaluation with younger peers (n = 35) Brief open exit slips (n = 300 slips) with younger peers before and after their HPV vaccine offer

In Phase 1, qualitative focus groups (n = 32), comprising seven focus groups, were held with available teenagers who volunteered in their small working groups to evaluate their overall experiences, knowledge, confidence, and advocacy for the HPV vaccine at the completion of Phase 1 by CGB and EM. Focus groups are appropriate for exploring and debating topics and can reduce power dynamics.^31^ A topic guide was used to cover participants’ experiences, HPV knowledge, and suggestions for future workshops, which lasted between 10 and 45 minutes (Table 3).

**Table 3.** Qualitative topic guides and prompts.

Teens
**Topic Guides for focus groups with teens (end of Phase 1)** Experiences of the STEAM intervention overall Knowledge of immunology, vaccines, HPV and cancer-prevention since before and after Phase 1 Confidence in telling others about the HPV vaccine Future suggestions for Phase 1
**Topic Guide for focus groups with teens (end of Phase 2)** Experiences of the Peer Education/presentations Views of younger peers learning Views of the presence of experts Confidence in telling others about the HPV vaccine Future suggestions for Phase 2
**Younger Peers**
**Qualitative in-class evaluation with younger peers [After Peer Education and Pre-Vaccine offer]** Knowledge recall from the Peer Education Q&A
**Open exit slips Phase 2 with young peers [After Peer Education and Pre Vaccine offer]** “If you were to tell a friend who was absent today about HPV and the vaccine, what would be the main points that you’d highlight for them? Think about it for a moment then write the keywords that you would include with your summary for them and, if you want, draw a picture to accompany them.”
**Open exit slips Phase 3 with young peers [After Peer Education and Post Vaccine offer]** Knowledge recall from Peer Education Q&A

In Phase 2, qualitative focus groups (n = 21) were held with available teenagers in five focus groups in their small working groups to evaluate their experiences of Phase 2 by CH and IM using topic guides (Table 3). Qualitative in-class evaluation also took place in the primary school in situ for younger peers (aged 11–12 years) (n = 35) to consider knowledge recall, and open exit slips were also used to collect these data at secondary schools (aged 12–13 years) (n = 275 slips), facilitated by CH and IM prior to their HPV vaccine offers.

In Phase 3, open exit slips were also used in class during school visits with university experts to consider knowledge recall after younger peers’ HPV vaccine offers (12–13 years) (n = 25) after a longer period, although this phase was limited. A Miro board was used as a digital space to manage and organize open exit responses, and affinity mapping was used to identify themes.

### Analysis

Qualitative data from the focus groups and in-class evaluations were audio-recorded and transcribed verbatim. The analysis was informed by Braun and Clarke’s reflexive thematic analysis, comprising a six-phase framework that ensures familiarity with the data, generation of initial codes, searching and reviewing into themes, defining and naming these, and report-writing.^32,33^ Reflexive thematic analysis is iterative and relies on data verification through discussion of nuances. This is suitable for addressing the pragmatic aims of this study and is an interpretative approach. Coding of focus groups and in-class evaluation was initially conducted inductively by CGB and discussed with team members (CH, IM, EM, and RF) to refine these into meaningful interpretative themes. Open exit slip data were thematically mapped by AM and then synthesized by the team in the overall analysis. Robust systematic processes were adopted to analyze the results, as detailed above. Multiple qualitative data collection methods were used to evaluate the STEAM intervention, including focus group discussions, in-class evaluation, and open exit slips over 15 months duration. The method choice was pragmatic around acceptability for participants, timing, and minimizing school disruptions. Triangulation of different datasets adds to the credibility of the results.

### Ethics

This study was approved by the Maynooth University Ethics Board (SRESC-2022–2485559). Edinburgh Napier University’s Research Integrity also provided approval following the lead institution’s approval. All participating schools provided their consent to participate. Informed written consent was obtained from all participating principals of secondary and primary schools, relevant teachers, parents/guardians, teenagers, and younger peers, who were provided with tailored information sheets and consent forms. During the informed consent process, all participants read the tailored information sheets, were given a chance to ask any questions, and then signed a consent form to participate (Phase 1 and 2). Following consent and approval from school principals, we adhered to school management processes and guidance for the collection of voluntary exit slips from young peers (Phase 3). This study was conducted in accordance with the principles of the Declaration of Helsinki.

## Results

### Teenagers’ increased HPV knowledge, confidence and advocacy

We analyzed our qualitative focus group data with teenagers cumulatively (Phases 1 and 2). An overarching *egalitarian* theme centered on a sense of being equal in teenagers’ participation in the STEAM intervention through democratic processes, cited as unique. We identified three main themes from the reflexive thematic analysis surrounding this overarching theme: *1) Being out of your comfort* zone, which referred to navigating new and sometimes uncomfortable experiences; 2) *Growing HPV knowledge and awareness* which saw perceptions of increased knowledge and understanding through the intervention; and 3) *Being trusted vaccine advocates* which referred to perceptions of increased confidence in being able to tell others about this new knowledge in Phase 2 and outside the STEAM intervention. Illustrative data are presented below, along with codes for the phases and datasets.

#### ‘Being out of your comfort zone’

Teenagers reported overall positive experiences with their STEAM participation which could sometimes be uncomfortable; they cited facing unfamiliar situations, including new places, people, ways of working, and expectations that pushed teenagers outside their comfort zones but in manageable ways. Physically, teenagers worked on a university campus with students from other schools and university teachers (Phase 1) and co-facilitated workshops for younger peers in primary schools or their own secondary schools (Phase 2), rather than working solely at their usual schools. They contrasted this experience with that of the school, both physically and psychologically. Being physically present on a university campus was highly rated as an experience of being able to see campus life and work with university staff; many had never attended a university before. Psychologically, the experience of working with university staff was contrasted with that of school staff when probed.

TY6: I just think it’s kind of different from school because you kind of got to know you kind of better, and it wasn’t as serious as a classroom based […] We didn’t feel that we were like, that you were in charge of us, that you kind of gave us like, creative freedom when we were doing the activities.

EM: And that’s a little different from school, is it?

TY3: You know you walk into a classroom; you kind of know all the teachers. The teacher is above you like, know what I mean, but when you’re walking in here, obviously, yes, you’re above us, but you make us feel like we can just talk to you […] (Phase 1, FG 6)

Participants contrasted the STEAM experience with their usual classroom experience around hierarchical structures at school, compared to the approachable and democratic way of working with university staff, as seen above. Working with new people in allocated groups (teenagers from other schools) and university staff could feel daunting at first. New ways of working supported overcoming feelings of initial nerves and working on new topics. Teenagers rated approaches, including drama, role-play, drawing, and small group work on posters and small presentations, as useful to collaborate with other teenagers and university experts. They regarded these approaches as being able to facilitate learning effectively in chunks. For example, new knowledge was facilitated through short interactive sessions on HPV, vaccines, immunology, and the immune system delivered by experts. New skills, such as presentation skills, were developed over time in the small and larger group work (Phase 1), and presentations were then refreshed and co-developed (Phase 2), culminating in presentations to groups of younger peers. New learning was facilitated through the creative and film-making experiences combined with some of these new approaches which were seen as ‘fun.’ This overall common STEAM experience was summarized by one participant:

TY3: There’s a lot of things [to] take home, over how enjoyable it was, it could be challenging at times with the tasks, and you know, trying to meet new people to do videos with them for the presentation obviously for going round with primary schools. I think when you look at all the different facts that go into the work […] It’s part of the challenge and very enjoyable anyway. You know to come to such a large institution like Maynooth [University] and do a bit of research and have good craic [fun], same time it was something different, you know out of your comfort zone. (Phase 1/FG7).

New expectations were highlighted in the STEAM intervention, and, initially, some participants reported being unclear about their roles at the start of the intervention, which became clearer through participation and time. Ultimately, participants worked to educate/act as peers for HPV which was out of their usual experience.

#### Growing HPV knowledge and awareness: ‘I didn’t know what HPV was’

Teenagers perceived that their overall knowledge of HPV, vaccines, immunology, and cancer prevention had increased compared to the start of Phase 1. For example, teenagers were asked to reflect on their HPV knowledge and any changes to it after the workshops (Phase 1):

TY5: Yeah, I think it’s [knowledge] changed, cause going in, I just knew it was some sort of virus. That I didn’t know anything really, like there was 150 different types or anything that was causing cervical cancer. So, I feel it’s better now that I know more about it, so that when we got to the primary schools, that if someone asked me a question. I’d be able to answer better than I could before.

TY2: Yeah, I definitely know more now than I did when I was coming in. Like I didn’t know first thing […] Now like I know loads, so I can tell my friends better and then they go to their friends or whatever […] (Phase 1, FG4).

The social aspects rather than the instructional aspects of learning were also highlighted and growing awareness of disease severity. For example:

TY7: We learned a lot more about it [HPV], like in I barely knew anything and you ‘oh well it’s a disease and it’s not good for you,’ and then that was it pretty much. But now, with knowing a lot more about it that we finished the workshop and the all the videos and the projects and that.

TY6: I didn’t know how dangerous it was until we did this […] But then when you hear like it can cause up to six types of cancer and then like it can like just one vax, not getting one vaccine can change your whole life […]. (Phase 1, FG3).

Significantly, teenagers reported knowledge of the wider HPV-associated cancers that could affect both men and women, in keeping with the national HPV vaccine policy to offer the vaccine to both boys and girls and their changing roles in educating others. For example:

TY2: […] I was talking with my parents and my Dad said, a guy, boys couldn’t get it [HPV vaccine]. Now, it’s here me and my mam are like no; they do because it was after the first workshop.

CGB: So, you were kind of educating your Dad?

TY2: Yeah, it’s a change, instead of him educating me (Phase 1, FG 3).

#### Being trusted vaccine advocates: ‘I would tell people now’

Expanding on the above, this theme related to perceptions of being a vaccine advocate with increased confidence gained throughout the STEAM intervention. Teenagers reflected on their peer education experiences (Phase 2). At times, the younger classes were seen as ‘shy’ or not interactive. Several integral aspects were highlighted for the successful implementation of peer education, including approaches to reach their audience using videos/films that often utilized comic affect to address a serious topic; being recognizable as older peers and thus seen as trustworthy; being prepared with scripts and refresher workshops; and having experts on hand to support the delivery. For example, teenagers reflected on their ‘hero’ like status with younger peers, given their ages, and that their messages and behaviors would hold greater weight compared to adults:

TY6: They [young peers] can’t wait to be us. Like, they look up to us. But like so, in a way they’re kind of like ‘if they got the vaccine, it’s obviously doing something right […] and they talk so confidently about it, and they’re so positive about it and they’re like they stand for so much, like so obviously it’s good.’’

TY2: Yeah, there’s more chance they’re gonna go back to their parents and say ‘oh yes, and TYs came into to talk to us’ rather than it was just some like boring lecture from like an adult (Phase 1, FG3).

Compared to the start of Phase 1, teenagers reported that they felt more able to tell others about the HPV vaccine with growing confidence and understanding. Teenagers felt more able to speak to others in wider social groups (e.g., family and friends). For example, after delivering peer education in primary schools, teenagers were asked about their confidence in telling others about vaccines:

TY1: I feel relatively confident, like I’ll tell say, like family members or friends about it and recommend it.

CH: Ok, and would you have felt confident before you started this?

TY1: Definitely not. I wasn’t even sure what it [HPV] was to be honest […].

TY2: I didn’t know what it stands for, and I didn’t know, like that the other facts are like cancer or like vaccines and that. I didn’t know any of that. So, I wouldn’t haven’t told anyone […]. I would tell people now. I would, like I know the name the information to tell them, just so they know a bit more about it as well. (Phase 2/FG1 from primary school 1).

Increasing confidence in being able to tell others about the HPV vaccine was reported in all groups, although this varied, ranging from telling their close social networks to telling wider groups of known and unknown younger peers in formal and informal ways. Despite their increased confidence in formal presentations, teenagers reflected that they needed the support of university experts. For example, when asked how helpful it was to have experts present:

TY3: Very helpful. Because if we didn’t have you, and we didn’t know, and we blagged on something, and we were completely on our own, can’t rely on the primary school teachers to help us. They might not know anything about it.

IM: So, you were relying on us for what – our scientific knowledge?

TY3: It’s kind of the confidence factor of you being there. It’s like you don’t need the core thing (a rope) that helps you when you’re going rock climbing […] you’re better to have it and not need it than need it and not have it at all. (Phase 2/FG2/Primary School, 2)

Having experts on hand was deemed important, even if not needed, to support teenagers in their roles as peer educators, as seen above in the metaphor of rock-climbing and safety ropes. The teenagers recognized that they might not be able to rely on primary school teachers for subject-specific knowledge and support on this topic.

Teenagers also considered that following peer education, younger peers could potentially be able to educate others about HPV in the future. For example, by informing parents about HPV:

CH: What do you hope that [Primary School] got from it [Peer education]?

TY2: I hope that they, now they know what it is [HPV and the vaccine] that they can make up a decision, just like when talking to their parents, and not their parents just signing it and being like, ‘oh yeah, go ahead it’s good for you,’ because your parents, they probably don’t really know either. So now they can tell their parents about it as well.

TY1: […] Yeah. It was like that for me anyways, like I gave them [parents] the form, they signed it, handed it in, and I didn’t know a lot about it. (Phase 2/FG1 from Primary school 1).

This ideal imagined informed choice scenario, in which the teenagers expected their younger peers to have given their increased knowledge about HPV, appeared in stark contrast with their own reported past experiences of HPV vaccine decision-making with their parents, which was characterized by limited HPV knowledge from both parents and adolescents.

### Teenagers’ perceptions of younger peers’ knowledge recall and younger peers’ increased HPV knowledge

Tied to the above, teenagers reflected on their perceptions of younger peers’ increased knowledge following peer education (Phase 2) and were surprised at how much peers recalled and at their own abilities. They reflected on their rapport with the audience and considered it important to be trusted. Reaching the audience through videos was perceived to be more effective in attracting audience attention. Teenagers reflected on peers’ knowledge recall; for example:

TY1: Yeah. They did learn a bit, I could tell from the start that they didn’t know about much, but yeah, at the end they were able to talk through about a couple of things so yeah.

TY2: They knew a lot of stuff, I didn’t think they’d like, learned that there be like 150 types [of HPV] and there were 14 that were dangerous. I didn’t think that they’d remember that. But they did. (Phase 2/FG 1/Primary school 1).

Qualitative in-class evaluation (n = 35) was conducted in situ following peer education, which appeared to support teenagers’ perceptions that younger peers had increased knowledge of HPV and could recall pertinent information, for example:

CH: Tell me what do you know about the HPV vaccine that you didn’t know before now?

1: It can cause cancer

CH: Yeah, that HPV can cause cancer. Yeah, some forms of it are harmless, but some forms cause cancer. But there’s lots of types of HPV.

2: And there’s lots of types of it, but there’s 14 that can kill you. (Qualitative in-class evaluation with primary school).

Younger peers recalled some pertinent facts, once prompted, following peer education, as seen above, including the timing of their vaccine offers. Overall, they reported enjoyment of peer education, the videos, and suggested future information about transmission. Similar responses were observed in the open exit slips (pre-vaccination offer), which were used to assess younger peers’ knowledge (n = 275) in secondary schools (Phase 2). Through thematic mapping, key themes were identified: awareness of HPV, HPV virus and links with vaccination, HPV vaccine and cancer prevention, understanding of the vaccine process, positive attitudes toward the HPV vaccine, negative attitudes toward the HPV vaccine, and finally, transmission of HPV. Table 4 presents the key themes and their respective frequencies.

**Table 4.** Theme mapping across all responses (n = 275) from open-ended exit slips from younger peers in response to key messages.

Themes	School 1 Group 1	School 1 Group 2	School 2 Group 1	School 2 Group 2	School 2 Group 3	School 2 Group 4
Awareness of HPV	X	X	X	X	X	X
HPV & vaccination link	X			X	X	
HPV and Cancer prevention	X	X	X	X	X	X
Understanding the vaccine process	X	X		X	X	X
Positive attitudes to the HPV vaccine	X	X	X	X		X
Negative attitudes to the HPV vaccine		X	X			X
Transmission of HPV		X	X	X		

All groups showed some knowledge of HPV and the types of HPV that could lead to cancer. Three groups showed an understanding of the protective benefits of HPV vaccination. All groups showed an awareness of the HPV vaccine and its role in cancer prevention. Five groups showed some understanding of how the vaccine works, including the timing of administration and its function in the body. Five groups demonstrated positive attitudes toward the HPV vaccine. Three groups showed some negative attitudes toward the HPV vaccine regarding nervousness (the needle) and its discomfort. Three groups had knowledge of HPV transmission. Open exit slips (n = 25) were also used in secondary school after younger peers’ HPV vaccine offers to assess their knowledge recall in Phase  3 school visits after a longer period. These slips indicated persistent knowledge about HPV, the vaccine, and prevention of cancer (16/25 64%); the videos were most cited as being conducive to learning which supported teenagers’ own perceptions as reported above.

## Discussion

We qualitatively evaluated the effect of our STEAM Peer Education intervention on teenager’ knowledge, confidence, and advocacy for HPV communication with younger peers facing their HPV vaccination offers and younger peers’ knowledge recall. Our overarching egalitarian theme underpinned a democratic learning environment for teenagers characterized by learner autonomy, holistic learning, and a teaching environment to enable co-design of working and creative dialogic approaches to support teenagers’ confidence as HPV advocates through creative outputs and presentations. Our theme of ‘being out of your comfort zone’ emphasized a unique STEAM opportunity provided to teenagers to develop increased knowledge of HPV, immunology, vaccines, and cancer prevention and support which ultimately led to them acting as peer educators. Teenagers’ perceptions of their increased knowledge and sense of increased confidence in being able to tell others about the HPV vaccine were apparent in our analysis, compared with at the start of STEAM. Our inductive analysis is appropriate for this exploratory study.^34^ Theoretically, we considered our themes against the COM-B model, which theorizes that behavioral change (behavior as a vaccine advocator) requires the activation of three components: opportunity, capability, and motivation.^35^ Teens increased their physical and psychological capabilities in several aspects that were required for this behavior, including group and teamwork, posters and creative work, presentations, and knowledge about the immune system, HPV, vaccines, and cancer prevention. The STEAM intervention provided a unique opportunity for teenagers to participate in co-design work with multidisciplinary university experts on campus and in schools. Increased internal motivation to tell others and positive attitudes toward vaccination were also apparent, which is essential for behavior as a vaccine advocate. While increased confidence was apparent in all teen groups, not all teens felt confident in delivering peer education outside the STEAM intervention. This aspect should be further explored in future research.

Our STEAM intervention supports the evidence base on the use of peer educators for vaccines combined with health experts, but it is novel and extends this to multidisciplinary experts, including scientists, educationalists, and designers.^17^ Evaluations of interventions aimed at older teenagers (15–17 years) for increased HPV knowledge and vaccine uptake are limited by a lack of stakeholder engagement, a limited evidence base, and a focus on female-only cancers.^12^ Our STEAM intervention adds to the evidence base for the use of STEAM peer education by focusing on cancers affecting both men and women. The duration of the intervention over 15 months allowed interactive contact over a longer period to scaffold teenagers’ knowledge and skills, so it could be retained beyond a one-off time point, where activities in Phase 1 supported the development of knowledge in Phase 2.^12,15^

The results of our analysis with both teenagers and younger peers in the focus groups (teenagers), in-class evaluations (younger peers), and knowledge recall slips (younger peers) indicated that younger peers were able to recall pertinent knowledge regarding HPV and cancer prevention. Specific knowledge can be measured in future surveys. Some younger peers indicated that they wanted more information about transmission and worried about needles/discomfort, which has been previously reported [for example].^36^ Both teenagers and younger peers considered videos effective for learning in this age group.

## Limitations

We did not have ethics approval to collect HPV vaccination data. Future research could investigate the effects of HPV vaccination rates following the intervention, along with changes in HPV knowledge and confidence in advocacy, by using a survey design. One school dropped out of the intervention after Phase 1, an all-boys school. It is important that boys be included in future HPV interventions, given the wider messaging needed to target male cancers.^12,13^ The scalability, feasibility, and resourcing of this STEAM intervention can be considered in other contexts. This study took place in one setting in Ireland; we make no claims to the generalizability of the results to other settings.

## Recommendations for peer education

- Multidisciplinary STEAM approaches, rather than health-only expertise, may be effective in addressing HPV vaccine hesitancy and can be explored in other contexts.
- Older peer educators still needed the support of university experts for confidence
- Beyond the effects of the intervention, peer educators differed in confidence in their vaccine advocacy. This aspect should be explored in future studies.
- Longitudinal interventions may allow learning and scaffolding to occur when supported by experts and enable teenagers to model learning approaches with their younger peers.
- Older peers considered their positive behavior (vaccination) and communication to be influential with young peers, and this should be explored among younger peers.
- Older peers considered that parents would be educated by the younger peers, and changing parental attitudes and knowledge should be evaluated in future studies.

## Data Availability

To preserve the privacy of individuals, raw data (recordings and associated raw transcripts) are not publicly available. An aggregated summary of the data results and STEAM intervention approaches may be available upon request from the authors.

## Note

1. The all-boys school participated in Phase 1 education and co-design but later withdrew from subsequent phases.
